# Fragility Fractures of the Pelvis—Current Understanding and Open Questions

**DOI:** 10.3390/jcm14145122

**Published:** 2025-07-18

**Authors:** Amber Gordon, Michela Saracco, Peter V. Giannoudis, Nikolaos K. Kanakaris

**Affiliations:** 1 Leeds Major Trauma Centre, Leeds Teaching Hospitals, Leeds LS1 3EX, UK; amberg3@ortho.arizona.edu (A.G.); michelasaracco@gmail.com (M.S.); pgiannoudi@aol.com (P.V.G.); 2 Academic Department of Trauma and Orthopaedics, University of Leeds, Leeds LS2 9JT, UK; 3 Department of Orthopaedic Surgery, University of Arizona, Tucson, AZ 85721, USA; 4 Department of Public Health, Division of Orthopedic Surgery, University of Naples Federico II, 80138 Naples, Italy

**Keywords:** pelvic fractures, fragility fractures, FFP, insufficiency fractures, low-energy trauma, elderly, osteoporosis, comprehensive review, reconstruction ladder

## Abstract

Fragility fractures of the pelvis (FFPs) are common in elderly patients, particularly those with osteoporosis. FFPs can be associated with high mortality, morbidity, and functional decline. Known risk factors include being over 80 years old and delays in surgical intervention when this is required. While the role of surgery in FFPs remains less defined than in proximal femoral fractures in the elderly, studies indicate that surgical fixation offers improved survival and functional outcomes. Similarly, the choice of fixation method, whether posterior or anterior, and their combinations, vary between clinicians. It depends on the fracture type and patient-specific factors, such as bone quality and comorbidities, as well as the surgeon’s experience and the availability of resources. Additionally, orthobiologic adjuncts such as cement augmentation and sacroplasty can enhance the stability of an osteoporotic fracture during surgical intervention. Furthermore, medical treatments for osteoporosis, especially the use of teriparatide, have demonstrated beneficial effects in reducing fractures and promoting healing of the FFPs. Return to pre-injury activities is often limited, with independence rates remaining low at mid-term follow-up. Factors that influence clinical outcomes include fracture type, with Type III and IV fractures generally leading to poorer outcomes, and patient age, functional reserve, and comorbidities. The present tutorial aims to summarise the relevant evidence on all aspects of FFPs, inform an updated management strategy, and provide a template of the reconstruction ladder referring to the most available surgical techniques and treatment methods. Further research, based on large-scale studies, is needed to address the open questions described in this manuscript and refine surgical techniques, as well as determine optimal treatment pathways for this vulnerable patient population.

## 1. Introduction

Fragility fractures of the pelvis (FFPs) represent an increasingly important concern in contemporary orthogeriatric care. Over the past decade, the prevalence of FFPs has risen steadily, reflecting the growing ageing population and the associated burden of osteoporosis [1,2].

Osteoporosis is a very common bone disease, known as the “silent” disease, that exposes individuals to an increased risk of fractures. This is due to a decrease in bone mineral density and bone mass, leading to a decline in bone strength. Fragility fracture of the hip is the most common type of fracture of the lower extremities associated with this pathological condition, but it is not the only one [3].

Osteoporosis-related fractures generate high social costs and are associated with a high risk of mortality and morbidity. Prevention is therefore essential through maintaining a healthy lifestyle and engaging in regular physical activity. Screening tests for early identification of reduced bone density are essential to start appropriate pharmacological and non-pharmacological therapies [4].

Due to reduced bone density and compromised osseous architecture, FFPs predominantly affect elderly individuals, particularly women. Epidemiological data underscore the importance of addressing FFPs, as the incidence escalates significantly with age, with most cases occurring in individuals aged 65 years and older [5,6,7].

The impact of FFPs extends beyond the initial injury, contributing to considerable morbidity and mortality. Mortality rates for these fractures can reach as high as 27%, and only a minority of patients regain sufficient mobility and independence to return to their own homes [8,9,10]. This highlights the critical need for timely diagnosis, comprehensive assessment, and effective intervention strategies to improve the outcomes of affected patients. Early recognition and management of FFPs can mitigate their profound effects on functional independence and quality of life [10,11].

This paper examines the definitions, epidemiology, risk factors, diagnostic approaches, and classification systems related to FFPs. Through a detailed analysis, this study seeks to provide insights into optimising clinical care and advancing strategies for managing this increasingly prevalent condition in an ageing population. Furthermore, it aims to highlight areas of high importance and open questions that should be the focus of further clinical research.

## 2. Materials and Methods

A comprehensive review of peer-reviewed publications was conducted using the databases of PubMed, Medline, and Scopus, focusing on recent studies related to fragility pelvic fractures. Keywords included “fragility pelvic fractures,” “osteoporosis and pelvic fractures,” “diagnosis,” “management,” and “treatment”. Inclusion criteria encompassed original research articles, review papers, and clinical guidelines published in the last 10 years. Articles were excluded if they primarily addressed high-energy pelvic fractures or referred to non-elderly patient cohorts.

## 3. Definitions/Epidemiology

These fractures are defined by consensus as fractures of the pelvic bones resulting from either low-energy trauma (usually simple falls) or stress fractures without a history of trauma (insufficiency fractures) [5,11].

Given the epidemiological data, FFPs are commonly diagnosed in patients aged 65 years or older, capturing a significant portion of the population most affected by these types of fractures. The age threshold of 65 years, particularly among females, aligns with the general trend of fragility fractures, which are fractures that occur from minimal trauma that would not typically result in a fracture in a healthy individual. The incidence of FFPs increases significantly with age, with the average age of occurrence ranging from 80 to 90 years [10,12].

The incidence of pelvic fractures in patients older than 85 years has been reported to be 450 cases per 100,000 population, compared to 37 cases per 100,000 population in the general population. [13,14]. The prevalence of FFPs has steadily increased and continues to rise over the past decades [15,16,17,18].

### Risk Factors

The most significant risk factor for FFPs is osteoporosis. Osteoporotic bones have a compromised osseous architecture compared to non-osteoporotic bones. This weakened structure predisposes individuals to fractures, which can occur even without trauma (insufficiency fragility fractures due to repetitive stress) [11,19]. Another notable risk factor for FFPs is the history of operative treatment for associated fractures, particularly of the proximal femur (e.g., hip fractures) and/or multisegmental surgery to the lumbar spine [7,20]. High pelvic incidence or residual unmatched lumbar lordosis leads to increased transmission of stresses to an osteoporotic sacrum which can lead to FFPs [21,22].

According to Zoulakis et al. [23], despite patients with type 2 diabetes mellitus (T2DM) having higher bone mineral density (BMD), they experience decreased physical ability and an overall increased fracture rate. Previous studies have indicated that inadequate glycaemic control is linked to an elevated risk of fractures in individuals with T2DM [24].

Another significant comorbidity to discuss, despite normal or high BMD, is obesity, which suggests that bone quality may be more critical than bone quantity. Evidence supports that while total body fat influences BMD, the distribution of adipose tissue plays a significant role. For instance, higher adipose tissue located in the viscera has a greater negative impact on BMD and fracture risk [23]. Obesity is also associated with an increased risk of falls due to adipose deposition in muscles, which leads to weakness and decreased balance.

Furthermore, sarcopenia, the age-related loss of skeletal muscle mass and strength, can also lead to a decline in physical function, increased risk of falls and fractures. It may also affect patients with obesity at any age. Sarcopenia, obesity or malnutrition, chronic diseases, and inactivity are interlinked as risk factors leading to poor quality of life and increasing the risk of falls and fragility fractures. Therapeutic weight loss, bariatric surgery, and physical activity allow the trend to be reversed, making these risk factors correctable [25,26].

In individuals with both obesity and diabetes, the risk of falls may be even higher. This dual risk is compounded by muscle weakness, balance issues, and potential neuropathy associated with diabetes. Interestingly, obesity and sarcopenia fall into the category of modifiable risk factors for fractures [24,27].

Another important modifiable risk factor to address is alcohol consumption. In a systematic review by Ke et al. [28], which included 44 studies covering 6,069,770 participants and 205,284 cases of fractures, it was concluded that any level of alcohol consumption is a risk factor for total fractures. Chronic alcohol use negatively impacts bone quality and increases the likelihood of falls due to its effects on balance, coordination, and muscle strength.

## 4. Diagnostic Approach

### 4.1. Clinical Presentation

Patients with FFPs typically present with pelvic pain, often without a history of significant trauma, but typically following a fall from standing height or an increase in pain over a period. This pain usually prevents patients from bearing weight as they typically would, significantly hindering their ability to perform daily activities. In some cases, patients may present days or even weeks after the injury for a variety of reasons, including initial misdiagnosis, delayed seeking medical care, or an inability to recognise the severity of their condition. This delayed presentation can complicate the clinical picture and delay appropriate treatment.

Prolonged immobility due to pain or the inability to bear weight may lead to secondary complications, including pressure ulcers, venous thromboembolism, muscle atrophy, and increased mortality. Physical examination findings, such as tenderness over the sacrum, iliac crest, pubic symphysis, or pain with pelvic compression testing, are not usually diagnostic. Local comorbidities (as hip or facet joint osteoarthritis, chronic arthropathies, spinal stenosis, trochanteric bursitis, and other hip conditions, etc.), but also cognitive impairment (recorded between 20 and 40% in the population of FFPs), commonly prevent the completion of a reliable clinical assessment. The reported symptoms of FFPs are similar to those of these pre-existing conditions, and differential diagnosis can be hindered and/or delayed [10,29]. Therefore, in almost all cases, radiological investigations are used to gather further information.

The risk of severe bleeding and associated hemodynamic instability, although less frequent in comparison to standard high-energy pelvic injuries (2.4% vs. 40%), should also be considered in patients with FFPs [30,31,32]. Due to the atherosclerosis of the vascular tree, lower cardiovascular reserves and the frequent use of anticoagulants in this population, they should be assessed and monitored for associated arterial lesions (most commonly reported at the anterior division of the internal iliac, obturator, superior vesical, inferior epigastric or superior gluteal arteries) and significant blood loss following even relatively benign pubic rami fractures [10,14,33].

### 4.2. Imaging

Early diagnosis of FFPs can significantly reduce the period of uncertainty and persistent pain and improve mobility in affected patients by guiding an appropriate management plan. When establishing a diagnostic algorithm for FFPs, several factors must be considered, including the accuracy of the diagnostic test, cost-effectiveness, and accessibility.

Plain X-rays are typically the first-line diagnostic tool in terms of cost-effectiveness due to their low cost and high availability. However, plain radiographs present challenges in diagnosing FFPs due to poor bone quality, interposing visceral shadows, and limited initial displacement of the fractures in many cases. Studies have shown that fractures of the posterior pelvic ring are often missed, with detection rates ranging from 32% to 87% [34,35]. Kanakaris et al. [10] reported that more than 80% of FFP patients with persistent pelvic pain end up having advanced imaging at some point in their care. Therefore, performing this early is sensible as missed FFPs can lead to persistent pain, immobility, and higher mortality rates [34].

A computerised tomography (CT scan) is instrumental in diagnosing FFPs as it provides high-resolution, cross-sectional pelvic images. Even in the presence of subtle, less displaced fractures of the posterior pelvic ring, it improves the accuracy (up to 90%) of diagnosis, which potentially influences treatment decisions [34,36].

**Figure 1:**
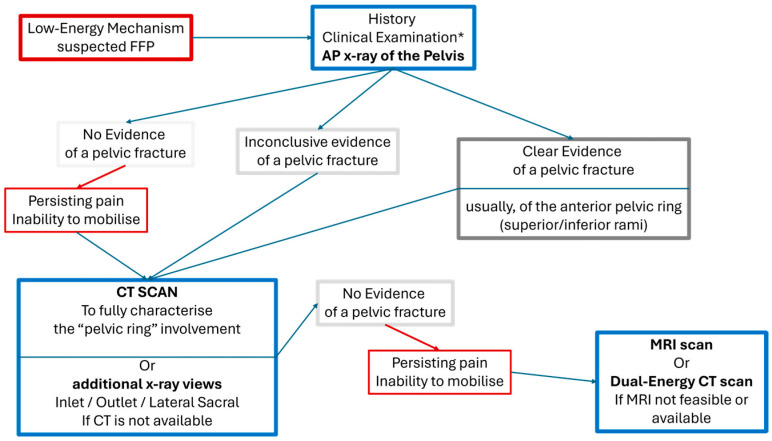
Diagnostic flowchart when suspecting FFPs. * Clinical Examination should include between others: deep palpation over the sacrum and the sacroiliac joints; flexion-abduction-external rotation stressing of the hip joints; axial loading of both femurs; straight leg raising tests.

The use of magnetic resonance imaging (MRI) and, more recently, dual-energy computed tomography (DECT), raises the accuracy close to 100%. In the presence of occult stress fractures and severe osteoporosis, the addition of these two modalities identified additional sacral involvement in up to 22% of the patients, who were recorded to have unilateral sacral fractures with a CT scan [36,37,38,39]. Despite the benefits, MRI poses challenges, including high costs and limited access. Currently, it is considered for patients with persistent symptoms despite normal X-ray and CT results or a history of malignancy. A simple diagnostic flowchart based on previously published evidence is described in Figure 1 [10,40].

### 4.3. Classification Systems

The main differentiator of FFPs from the pelvic fractures of the general population is the limited trauma to the surrounding soft tissue structures and initial displacement. Therefore, the general classification systems of pelvic fractures (Peltier—1951, Letournel—1978, Young Burgess—1986, Tile—1988, AO/OTA—1996), the pubic rami (Starr Nakatani—2008), and the sacrum (Roy Camille—1985, Denis—1988, Isler—1990, AO Spine—2020) are unable to characterise fragility pelvic fractures fully, (Table 1) [41,42,43,44,45,46,47].

In 2013, Rommens and Hofmann introduced the term FFPs and categorised them into four main types [46].

- FFPs type I: Stable non-displaced fractures that affect only the anterior pelvic ring (Ia lesion: unilateral; Ib lesion: bilateral).
- FFPs type II: Partially unstable and non-displaced fractures involving the anterior and/or posterior pelvic ring (IIa: unilateral and non-displaced sacral fractures; IIb: fractures of the pubic rami and the sacrum (ventral cortex); IIc: fractures of the pubic rami and complete (ventral and dorsal cortices) sacral fractures).
- FFPs type III: Unstable and displaced fractures with complete disruption of the pelvic ring. (IIIa: pubic rami and iliac wing fractures; IIIb: pubic rami and iliosacral crescent fracture/dislocations; IIIc: pubic rami and sacral wing displaced fractures).
- FFPs type IV: Highly unstable fractures, with complete dissociation between the spine and the pelvic ring. (IVa: bilateral iliac wing/crescent fractures; IVb: bilateral sacral fractures/U-shaped fractures with transverse sacral fracture line at S1/S2; IVc: different lesions between the two sides with combinations of trans-iliac/trans-sacral/transilio-sacral instability).

This classification system has been found to be valuable for guiding treatment decisions based on the stability and location of the fracture. Stable fractures (Type I) can be treated non-operatively, while Type II fractures often benefit from percutaneous screw fixation. For Type III fractures, ORIF is typically recommended, and Type IV fractures, due to their severity, require iliolumbar fixation or a combination of advanced surgical techniques. The Rommens and Hofmann classification has been generally adopted and validated over the last decade [15].

The OF-Pelvis classification, introduced in 2021 by Ullrich et al. [47], divides pelvic ring injuries into five subgroups with three modifiers based on findings from CT and MRI at the exact localisation. The subgroups categorize injuries by increasing severity, starting with OF1, which describes pelvic ring oedema visible on MRI without a fracture on CT. OF2 includes fractures of the anterior pelvic ring on one or both sides. OF3 involves unilateral sacral fractures, with or without anterior ring lesions, while OF4 describes bilateral sacral fractures, with or without anterior ring lesions. OF5 represents iliac or sacroiliac fractures, with or without anterior ring lesions. The three modifiers (M1-M3) relate to stability and specific features but are not hierarchical or indicative of increasing injury severity. M1 corresponds to L5 transverse process avulsion fractures, signifying disruption of the iliolumbar ligaments. M2 pertains to displacement, and M3 identifies additional localised oedema visible on MRI, confirmed by CT. These modifiers can be applied individually or in combination. The primary advantage of the OF-Pelvis classification is its comprehensive consideration of the entire pelvic ring. However, its main limitation lies in the limited number of classified cases available for study. Despite this, the authors of this classification system demonstrated acceptable inter- and intra-observer reliability, making it a promising tool for assessing and managing FFPs [47].

## 5. Management Strategies

The primary goals of treating FFPs are to enable patients to ambulate and heal the fractures, thereby avoiding secondary complications. The risks associated with immobility are well-documented and include pneumonia, venous thromboembolism, pressure ulcers, and deconditioning. These risks are particularly significant in frail patients with fragility fractures.

Following the proven model of standardised clinical pathways for elderlypatients with proximal femoral fractures (NOFs), creating FFP-specific management protocols appears sensible. Several initiatives, with priorities such as optimising the diagnostic process, effective pain management, geriatric and osteoporotic work-ups, and physiotherapy protocols promoting early mobilisation, have shown promising results [10,48,49,50].

Historically, non-operative management was the standard approach for treating FFPs. However, this strategy has been challenged following the significant progress made in the available methods of operative treatment and the evident increased mortality of this vulnerable population.

**Figure 2:**
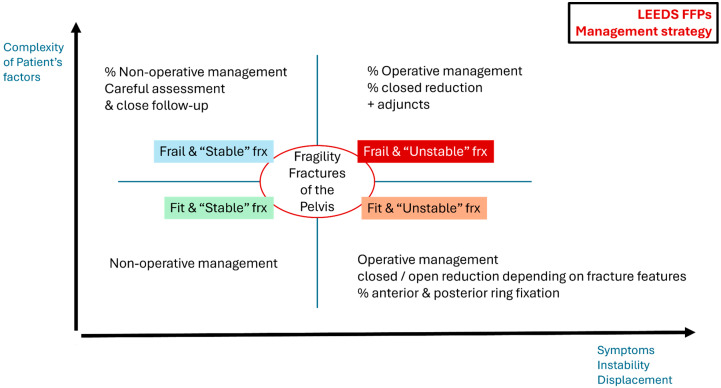
Representation of basic strategies in different types of patients of the general cohort of FFPs, depending on fracture complexity, symptoms, and instability, as well as patient-related factors as frailty and preinjury mobility.

The FFP classification provides a framework for determining treatment options, but more evidence is needed to establish a cost-efficient and outcome-relevant management algorithm. Several host parameters, including overall frailty, comorbidities, body habitus, pre-injury activity levels, and physiological state at presentation, should also be considered (Figure 2). Furthermore, standardised protocols regarding the timing of surgical intervention are needed. Swenson et al. [51] compared operative and non-operative treatments for FFPs, using pain as the primary outcome measure to determine whether there was a significant difference in outcomes. The surgical treatments assessed in this study included percutaneous sacral fixation, either iliosacral or transiliac-transsacral, with or without additional anterior fixation.

### 5.1. Non-Operative Treatment

Non-operative management primarily focuses on adequate pain control through prescription medications to facilitate early ambulation and prevent long-term immobilization. There is no universally accepted weight-bearing protocol; however, most treatment strategies involve restricted weight-bearing on the affected side, while some advocate for weight-bearing as tolerated [10,51,52].

Given the advanced age of this patient population, physical therapy plays a crucial role in promoting mobility. However, no standardised physical therapy protocol currently exists for non-operative management. Pain often serves as a limiting factor, and as described by Rommens et al. [15], failure to achieve mobilisation or adequate pain relief within 5–7 days may necessitate surgical intervention.

Additionally, patients managed conservatively require ongoing follow-up to monitor for potential progression of fracture displacement [10,15,49]. Surgical interventions in these cases are considered more challenging, as closed reduction becomes difficult. At the same time, open procedures are associated with increased blood loss, a higher risk of infection, and a longer hospital stay [53,54,55].

### 5.2. Operative Treatment

**Figure 3:**
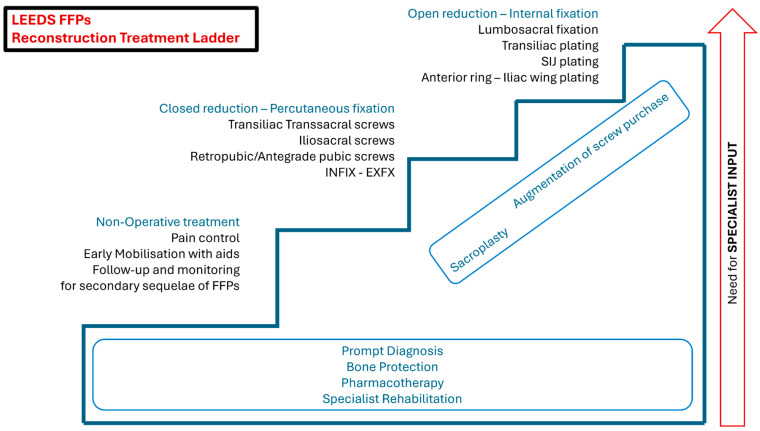
Graphic representation of different reconstruction options and the gradual escalation of interventions and specialist involvement required for the successful management of FFPs (Leeds Reconstruction Treatment Ladder).

Depending on the type of FFPs, surgical fixation of the posterior ring and/or of the anterior elements may be necessary (Figure 3) [46]. Since the posterior pelvic ring provides more than 60% stability while sitting or standing, its fixation is often prioritised [56,57]. However, there is no consensus or precise algorithm on the optimal fixation method, or whether addressing only the posterior elements is sufficient for some type II or III FFPs [58,59,60].

### 5.3. Posterior Fixation

Several techniques for posterior pelvic ring fixation, including percutaneous iliosacral, transiliac-transsacral screw osteosynthesis, transiliac bridge plating, lumbopelvic fixation, and bridging subcutaneous fixation (INFIX) have been described [15]. Among these, percutaneous screw fixation is widely accepted and associated with reliable outcomes. However, a key challenge in geriatric patients is the poor quality of fluoroscopic images due to the presence of poor bone density (especially in the presence of obesity) as well as the high risk of secondary loss of fixation due to screw loosening. This risk can be mitigated, if sacral anatomy and intraoperative imaging allow, by using multiple long screws that extend into the contralateral sacral ala [61] or even better to the ilium (transiliac-transsacral screw fixation) [62].

#### 5.3.1. Transiliac-Transsacral Screws

**Figure 4:**
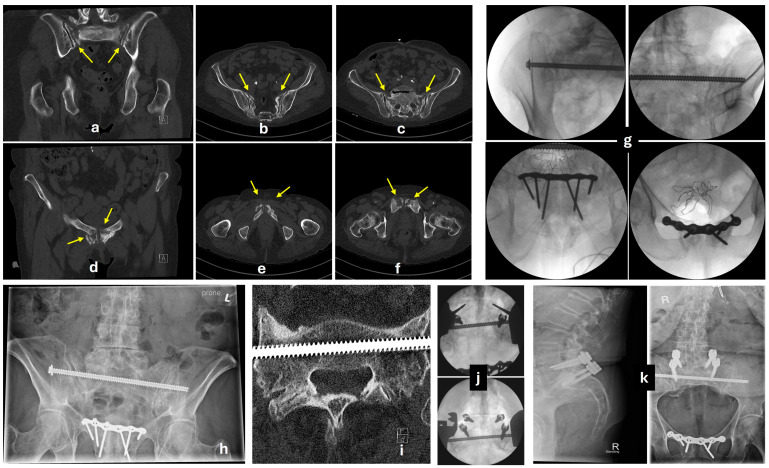
Case example of an insufficiency pelvic fracture. A 77-year-old female patient with severe osteoporosis, history of hysterectomy at a young age, bladder neck surgery, multiple fragility thoracolumbar fractures, was referred with worsening lower back and sacral pain for the last 5 months, without any history of trauma. MRI evidence of a high sacral H-type insufficiency fracture (transverse plane at S2). (**a**–**c**) CT-scan showing nonunion of the sacral fractures (yellow arrows), bilateral fractures of the L5 transverse processes, and (**d**–**f**) nonunion of the bilateral para-symphyseal fractures of the rami (yellow arrows). (**g**) Fluoroscopic views during the open reduction internal fixation of the rami fractures with a 3.5 mm reconstruction plate bridging the symphysis pubis and a single transiliac transsacral 8.0 mm position screw at S1 level. Full bone metabolic profiling was performed, and intravenous supplementation of her calcium and vitamin D levels was provided. She was allowed immediate mobilisation, weight bearing as pain permitted, with the use of a Zimmer frame. She gradually weaned off the frame and managed to mobilise with a single stick after the first two months. (**h**) Anteroposterior pelvic X-ray at 8 months. As expected, non-symptomatic loosening of the anterior fixation has occurred. At 12 months, she developed a new onset of severe back pain with additional neuropathic L5 nerve distribution. (**i**) CT evidence of complete union of the sacral fractures, no metalwork impingement. An MRI scan identified severe degeneration of the facet joints, bulging discs at L3/4 and L4/5 levels, stenosis of the right L5 neural foramen, and spondylolisthesis. (**j**) One year later, she underwent root decompression of right L5-S1 and L5-S1 fusion, with partial recovery of her neuropathic pain and improved mobility at the latest follow-up. (**k**) Anteroposterior and lateral X-rays at 12 months following the last procedure.

Transiliac-transsacral screws generally provide greater stability for unstable fractures. While they require a more complex trajectory, Hadeed et al. [63] found no significant difference in complication rates when comparing trans-sacral and iliosacral screws. Additionally, Goncalves et al. [64] demonstrated that a single transiliac screw has a biomechanical advantage over two iliosacral screws and even more advantage when two screws are used over one. This suggests that in elderly patients with poor bone quality, trans-sacral screws are preferable, given their biomechanical superiority and comparable complication rates (Figure 4).

Another point of discussion is whether fixation of an uninjured sacroiliac (SI) joint has any clinical impact. Heydemann et al. [65] found no significant difference in pain or function at least one year after instrumentation when comparing standard iliosacral screw placement across an uninjured pelvis.

The presence of a dysmorphic sacrum, as well as degenerative deformities and osteophytes or previous implants at the sacrum, is not an infrequent problem [66,67]. Careful preoperative planning of the fixation corridors with these techniques is of paramount importance [68].

Developments relevant to modifications of the standard percutaneous techniques [69,70,71], flexible fixation devices [72], and advanced imaging guidance [73] have been employed to avoid implant misplacement complications and optimise this surgical method.

#### 5.3.2. Transiliac Bridge Plating

Transiliac bridge plating is another minimally invasive technique for posterior pelvic ring fixation. Krappinger et al. [74], in 2007, noted that while it provides sufficient stability for unstable pelvic ring injuries, it carries risks such as nerve and vascular injury. Other disadvantages include the need for bilateral posterior incisions, limited reduction capabilities, the requirement for bilateral bridging of the sacroiliac joint in unilateral injuries, and an increased incidence of symptomatic hardware.

#### 5.3.3. Lumbopelvic Fixation

Lumbopelvic fixation is indicated in cases of spinopelvic dissociations (type C of the AO spine sacral classification system) [75,76], and in some type IV FFPS, malunion/nonunion surgeries, or when sacral fracture displacement and comminution necessitate increased stability. Several biomechanical and clinical studies have advocated for this more invasive method of surgery, which offers increased stability even in the presence of poor bone quality, as in the cases of FFPs [11,77,78].

### 5.4. Anterior Fixation

The involvement of the anterior pelvic ring in FFPs is frequent and mainly consists of fractures of the rami, and less frequently, ligamentous disruptions of the symphysis pubis. In only 10% of cases, it can be in isolation without any posterior ring involvement as previously reported [10]. This zone is usually the first to be diagnosed, even with plain X-rays, and although it receives less stress during routine activities, it can be symptomatic.

#### 5.4.1. Conventional Anterior Plating

Herteleer et al. [79] investigated whether conventional anterior plating is still a good option to treat anterior fractures in osteoporotic bone, comparing single and double plating in 48 patients. Revision surgery was performed in six cases. The screw loosening rate remained high due to bone quality, especially in multi-fragmentary fractures. Double plating was associated with a lower risk of failure. These advantages are negated by the invasiveness of this surgical strategy and proposed in cases of symphyseal disruptions, neglected, non-reducible, or malunited/non-united fractures [54].

#### 5.4.2. Percutaneous Screw Fixation

**Figure 5:**
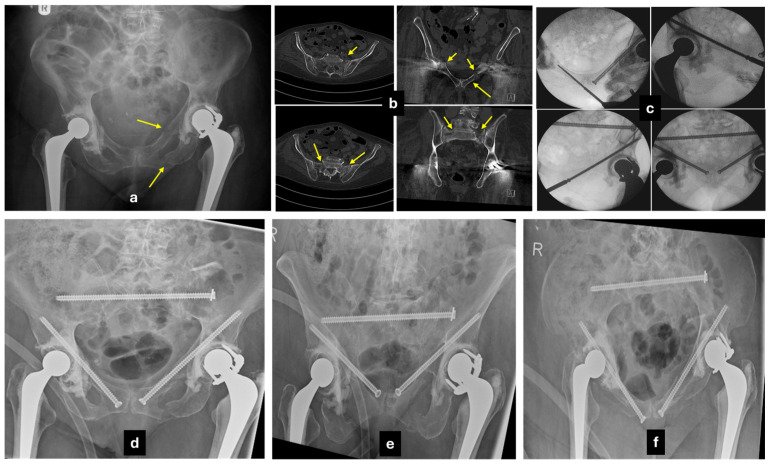
Case example of percutaneous fixation of both the anterior and posterior pelvic ring following an FFP-IIIC. This was a female patient, 86 years old, with a medical history of chronic kidney disease, atrial fibrillation, and bladder cancer, who presented following a simple fall. (**a**) Anteroposterior plain X-ray with left-sided superior and inferior rami fractures and suspicion of left sacral fracture. (**b**) Slices of CT-scan with clear identification of fracture planes (yellow arrows). The fracture was classified as FFP-IIIc. (**c**) Fluoroscopic views taken during the fixation of this FFP with two retropubic cannulated position screws, 6.5 mm, and one 8.0 mm transiliac-transsacral fully threaded cannulated screw, at the S1 level. The patient was mobilised without limitations postoperatively using a Zimmer frame for 6weeks. (**d**) Anteroposterior; (**e**) outlet; (**f**) inlet X-rays taken 13 months later with union and restoration of patient pre-injury mobility.

Percutaneous screw fixation of the anterior ring represents a very attractive option, but it requires surgical expertise, precision, and a reduced ramus (Figure 5) [80]. Acklin et al. [81] reported a biomechanical study on a cadaveric osteoporotic model about plating and screw fixation of the pubic ramus fractures. Not surprisingly, plate fixation was found to be superior to screw fixation regarding primary stability. In a biomechanical study, Arand et al. [82] investigated the combined implantation of sacral bars and anterior screw fixation for posterior and anterior pelvic ring fractures, type IIIc. The authors demonstrated a significantly higher stability with the addition of anterior screw fixation. According to Starr et al., percutaneous fixation was not associated with any clinically significant neurologic, vascular, or urological injury. However, the most common complication was loss of reduction, occurring in up to 15% of cases, with elderly patients experiencing the highest rate of fixation failure [80,83]. Photodynamic bone stabilisation systems and flexible intramedullary canal fixators have also been described for pubic rami fractures, with promising results and additional cost implications [72,84].

#### 5.4.3. External Fixator

The use of an external fixator for FFPs, despite its classic advantages of being less invasive and easily removed [85], is considered by most to be less well tolerated and even non-advisable [46], with a higher risk of loss of reduction and complications up to 62% [86].

Anterior subcutaneous two (INFIX) or three-point pelvic fixator has also been used to stabilise the anterior pelvic ring with a frame made of implants for spinal surgery [87,88]. In the supine position, pedicle screws are minimally invasively implanted bilaterally at the level of the anterior inferior iliac spines and, by some, also at the pubic body close to the symphysis (as a third point). The screws are connected by a bent rod running parallel to the inguinal ligament at the epifascial plane. These constructs were found useful to treat anterior fractures, especially in obese patients, avoiding the complications of external fixators and the risks of open approaches [88,89].

A variation in the minimally invasive rod INFIX was described in 2012 as a “pelvic bridge plating” [90,91]. A bridging low-profile contoured locking plate or a rod-plate construct (used for occipito-spinal fusions) is passed subcutaneously, spanning between the two iliac crests and anchoring to one or both pubic bodies. Cadaveric studies found the pelvic bridge to mirror better and therefore protect the vulnerable neurovascular anatomic structures, as well as being theoretically (absence of biomechanical studies between the two) a more versatile and stable construct with four points of fixation [92,93].

A posterior INFIX has also been described to stabilise the posterior pelvic ring, consisting of two pedicle screws placed in both posterior iliac crests, combined with a transverse rod crossing the midline of the posterior sacrum. In 2021, Muller et al. [94] published a systematic review about the results and outcomes of this technique, including eight papers with 186 patients. No major complications have been described apart from one surgical site infection, and four cases of implant loosening and good clinical outcomes were reported. Arand et al. [82] performed a biomechanical evaluation of an experimental INFIX versus standard screw fixation of a FFP Type IIIc fracture osteoporotic sawbone model. The extra-osseous bridging fixation was found to have significantly lower stability in comparison to standard intraosseous screw fixation.

### 5.5. Augmentation Techniques

Sacroplasty has also been employed in the management of sacral insufficiency fractures. This minimally invasive surgical procedure consists of cement injection through bone needles in the sacral wings using intra-operative CT or X-rays, like kyphoplasty or vertebroplasty. Singh et al. [95] reported significant pain relief within 24 to 48 h postoperatively. Schwetje et al. [96] proposed a balloon-assisted sacroplasty, reporting good results in 9 out of 10 cases. Kao et al. [97] tested a combination of long and short-axis alar sacroplasty, which required a longer operative time. The combination sacroplasty showed better results than the short-axis one, but it did not reach statistical significance.

However, as a fixation method is biomechanically less stable than screw fixation, making it a preferred option for nondisplaced insufficiency fractures. A known complication of sacroplasty is cement leakage, with an incidence of 10–20% as reported by Bastian et al. [98]. Potential adverse effects of cement leakage include delayed fracture healing, neurologic injury, venous embolism, and soft tissue irritation. Additionally, the presence of cement can complicate future fixation, as its removal is truly challenging.

Cement augmentation has also been proposed to reduce the risk of screws pulling out in osteoporotic bone, and it is often performed with sacroiliac screws implanted in fragility fractures. Biomechanical studies have described the positive effect of polymethylmethacrylate cement (PMMA) augmentation of cannulated iliosacral screws, leading to superior pull-out strength [99,100]. Augmentation can also be applied to transiliac and ilio-lumbar screws, as reported by Schmitz et al. [101], with good results reducing the screws loosening.

**Figure 6:**
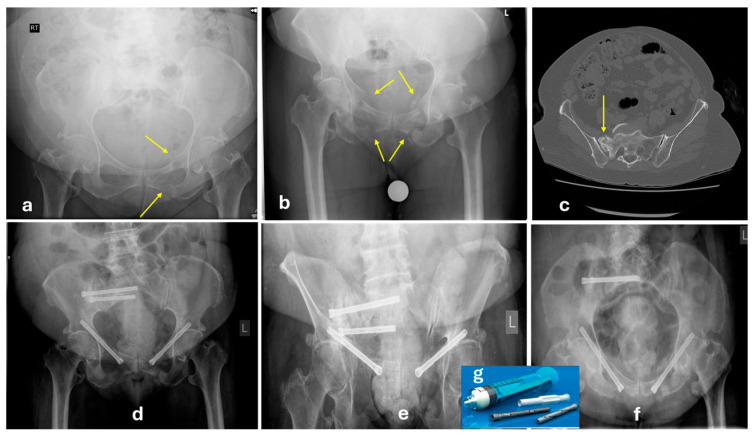
Case example of a 71-year-old female patient nursing home resident with a history of dementia, obesity, and rheumatoid arthritis. (**a**) Initial anteroposterior plain X-ray following a low-energy fall demonstrating fractures of the left superior and inferior rami (yellow arrows). Without further imaging (and with the limitations of the clinical examination due to her cognitive status and prior low back pain), she was diagnosed with an FFP-Ia stable unilateral anterior fracture and allowed to mobilise freely. No appropriate follow-up was organised, and the patient was discharged back to the nursing home. (**b**) Subsequent plain anteroposterior X-ray 8 months later, demonstrating fractures of all four rami (yellow arrows), incomplete healing of the initial left fractures, whilst she is now bed-bound and refusing to get out of bed due to pain. (**c**) Axial cut of a CT-scan with a Denis 2 type of sacral fracture with evidence of chronicity and nonunion (FFP-IIIc). (**d**) Anteroposterior (**e**) outlet, (**f**) inlet plain X-rays of the pelvis 3 months post-operatively following percutaneous fixation with bilateral retropubic fenestrated cannulated screws and left iliosacral screws at S1 and S2 levels augmented with calcium phosphate cement (**g**). The patient at this stage was mobilising unaided without complaining of pain.

Augmentation using calcium phosphate cement (CPC) has also been used with good results [101,102]. The use of CPC (Figure 6) avoids the risk of using non-absorbable materials, such as PMMA, which have poor handling features and the dangerous exothermic polymerisation phase. CPC augmentation has been tested to produce a 300% increase in the holding power of large fragment screws, with good resistance in compression but poor in shear forces [103,104,105,106,107].

### 5.6. Orthobiologic Adjuncts

Osteoporosis medical treatment is essential to treat patients affected by fragility pelvic fractures correctly and to prevent secondary fractures. Smith et al. [108] reported in their paper that, within 2 years, 41% of all enrolled patients developed additional osteoporotic fractures.

The strongest evidence in the literature for medical treatment of osteoporosis in patients with FFPs is reported for the use of parathyroid hormone (PTH) [10,109,110,111,112,113]. It should be administered only if the blood levels are correct, and calcium and vitamin D should be supplemented if necessary. The pivotal randomised trial of Peichl et al. [110] reported on a randomised trial involving 65 FFPs, testing the effect of a once-daily injection of 100 μg of PTH 1–84. They documented significant acceleration of the time to union, overall union rates, and better pain scores than the control group.

Novikov et al. [114] reported on the prescription of teriparatide (synthetic form of the natural human PTH) in patients with fragility pelvic fractures because of low rates of medical evaluation for its use in many cases or failure of insurance coverage in the United States. Several authors reported results about the administration of teriparatide in elderly patients with sacral insufficiency fractures. The authors demonstrated better outcomes with earlier pain reduction and mobilisation, resulting in a reduction in fracture healing time. Moon et al.’s [115] meta-analysis further demonstrated the efficacy of teriparatide in osteoporotic pelvic fractures, although the authors did not identify statistical significance advantages.

## 6. Reported Outcomes

### 6.1. Mortality

Mortality rates in elderly patients with NOFs are well established, up to 30% in one year, and significantly higher if treated non-operatively. Known risk factors include being over 80 years of age and a delayed surgery of greater than 48 h [116]. Studies on FFPs (similar population to the elderly NOFs) have shown that the one-year mortality rate ranges between 6.7 and 33% [10,15]. Rommens and Hoffman reported mortality rates of 6.9% on the operatively treated FFPs vs. 13.5% in nonoperative treatment [15]. Höch et al. [117] also showed that the mid-term mortality rate at two years was significantly lower in patients with surgically stabilised fractures (18% for those treated acutely operatively vs. 21% of those treated operatively after failure of nonoperative treatment vs. 41% of those treated nonoperatively). Despite the established mortality risk associated with FFPs, there is, in general, a lack of a systematic approach to developing appropriate FFP clinical pathways, similar to those for NOFs [10,49].

### 6.2. Return to Activities

Fragility fractures in general are associated with a high risk of complications and poor outcomes. This is also true in the case of osteoporotic pelvis fractures. Omichi et al. [9] reported a retrospective study on 552 patients with a mean age of 80 years old treated conservatively for pelvic, pubic, ischial, iliac, and sacral fractures classified by the Rommens classification (FFP). The subgroup analysis showed that the relevant difference in clinical outcomes correlated with the fracture type. Types III and IV were associated with worse survival rates and higher complication rates. The post-injury walking ability of the patients with Type III fractures was worse than that of the patients with Type I fractures. On the other hand, no significant clinical differences were demonstrated in the case of type II fractures, as reported by Zong et al. [118], between minimally invasive surgery and conservative treatment.

Kanakaris et al. [10] reported a recovery of preinjury mobility status at 12 months only for 41% of their 132 FFP cases, while this was statistically better for the subgroup of 67 FFPs that adhered to a specific diagnostic and treatment algorithm. Similarly, Hoch et al. [117] reported that only 26% of the enrolled patients could walk without aids after 1 year.

Nuber et al. [119] published an interesting prospective case-control study about clinical outcomes at mid-term follow-up of FFPs. A total of 154 patients were enrolled, and 53.2% underwent surgical procedures. Interestingly, the mortality rates were 10% vs. 25.3% in the operative vs. nonoperative groups, respectively. On the other hand, the complication rate was higher in the surgical group. In conclusion, the worst clinical outcomes and the highest mortality were seen in non-operatively treated patients.

The systematic review by Wilson et al. [120] documented improved mobility and function after surgery; however, its results were mostly hindered by the heterogeneous methodologies of the original studies it analysed. Walker et al. [121] showed significantly longer walking distances with operatively managed patients with transiliac-transsacral screws, who were also more likely to be discharged home (75% vs. 20%) compared to non-operatively managed patients.

Yoshida et al. [122] reported on 42 patients who underwent surgery with a mean follow-up of 12.8 months. A total of 35 patients were stabilised with percutaneous screw fixation, and seven required plate fixation (all type III and IV cases). They reported high rates (45.7%) of medical complications at the time of admission and implant loosening or backout. On the other hand, clinical improvement was observed, with good pain relief and only one surgical revision.

Oikonomidis et al. [84] reported using a photodynamic bone stabilisation system for pubic rami fracture fixation. At the 7-month follow-up, only 34% of the patients were independent. On the other hand, other authors [85,123] reported complete return to previous everyday life in most of their cases treated with variable methods, including supra-acetabular external fixators.

The clinical and functional outcomes of FFPS are strongly influenced by the patient’s age, comorbidities, functional reserve, type of fracture, and, of course, the specifics of the treatment method. The apparent discrepancies in methodology and terminology between the different studies, as well as the absence of large comparative studies, hinder the reach of conclusions. Determining which outcome measures are relevant to the quality of life of the elderly with FFPs is very difficult [124,125]. The high post-treatment death rates and the limited response rates due to cognitive impairment are challenging factors in all fragility fracture outcome studies. Moreover, the absence of FFP registries and specific care pathways, in contrast to those of elderly NOFs, creates additional challenges [126,127].

## 7. Conclusions

Over the last decade, the clinical entity of FFPs has been established as one of the main expressions of the orthogeriatric fracture syndrome. The era of conscious neglect of FFPs by clinicians and researchers has evolved into recent, intense efforts to define and classify them separately from the general pelvic fracture group, develop prompt diagnostic algorithms, and test and validate advanced treatment strategies, which include therapeutic regimes and specific fixation devices.

Each institution must introduce standardised FFP-specific clinical pathways, analogous to those for hip fractures, based on the availability of resources and local admission protocols. Prompt escalation of care, involving pelvic reconstruction specialists, should be facilitated for the subgroup of FFPs that are expected to benefit from surgery (Figure 5 and Figure 6).

Apparently, we are in a transition period during which numerous questions and dilemmas have been expressed by different research groups (Table 2), while clear answers remain limited at present. Many of the current solutions are based on the general literature on fragility fractures and the value of well-coordinated orthogeriatric care for elderly patients with fragility fracture-related problems (FFPs). The synchronisation between laboratory evidence and modern clinical research remains challenging, but also essential, as FFPs are expected to rise significantly in number.

**Table 1:** The FFP classification system and its correlation to other classification systems.

ROMMENS HOFFMAN [46] 2013	Description	OF-Pelvis ** [47] 2021	YOUNG BURGESS [45] 1986	Tile/AO/OTA [43] 2018	AO SPINE SACRAL [75] 2020	STARR NAKATANI [80] 2008	DENIS [41] 1988	ROY-CAMILLE [44] 1985	ISLER [42] 1990
**FFP I**	**only Anterior ring frxs**	OF2	n/a	61A2	n/a	zones 1/2/3	n/a	n/a	n/a
Ia	Unilateral	OF2	n/a	61A2.2	n/a	zones 1/2/3	n/a	n/a	n/a
Ib	Bilateral	OF2	n/a	61A2.3	n/a	zones 1/2/3	n/a	n/a	n/a
**FFP II**	**Undisplaced** **Posterior ring frxs** **+/− anterior frxs**	OF3	LC1	61B2.1	B	n/a	zones 1/2/3	n/a	(type a)
IIa	sacral frx without anterior ring frx	OF3	LC1	n/a	B	n/a	zone 1	n/a	(type a)
IIb	incomplete sacral frx with anterior ring frx	OF3	LC1 (stable)	61B2.1	B	n/a	zones 1/2	n/a	(type a)
IIc	complete sacral frx with anterior ring frx	OF3	LC1 (unstable)	61C1.3	B	n/a	zones 1/2/3	n/a	(type a)
**FFP III**	**Displaced Unilateral** **Posterior ring frxs** **+/− anterior frxs**	OF3/5	LC2	61B/C	(B)	zones 1/2/3	(zones 1/2/3)	n/a	(types a/b/c)
IIIa	Iliac wing frx	OF5	LC2	61B2.2	n/a	zones 1/2/3	n/a	n/a	n/a
IIIb	Iliac wing frx involving the SIJ (crescent)	OF5	LC2	61B2.2	n/a	zones 1/2/3	n/a	n/a	n/a
IIIc	Sacral frx	OF3	LC1	61C1.3	B	zones 1/2/3	zones 1/2/3c	n/a	types a/b/c
**FFP IV**	**Displaced Bilateral** **Posterior ring frxs** **+/− anterior frxs**	OF4	LC1-2-3/VS	61B/C	(B/C)	n/a	zones 3a/b/d	(types I/II/III)	types a/b/c
Iva	Iliac wing frxs	OF5	LC2	n/a	n/a	n/a	n/a	n/a	n/a
Ivb *	Sacral frxs	OF4	LC1/VS	61B3.2/C3.3	C	n/a	zones 3a/b/d	types I/II/III	types a/b/c
Ivc *	Combinations of posterior frxs	OF5	LC3/VS	61B3.1/C3.2	B/C	n/a	zones 3a/b/d	types I/II/III	types a/b/c

FFP: fragility fracture of the pelvis; frx/s: fracture/s; LC: lateral compression; n/a: not applicable; OF: VS: vertical shear. *: similar to the jumper’s fractures of young adults—displacement is often less impressive as the iliolumbar and iliosacral ligaments are still intact. **: Additionally, there are three modifiers (M1–M3) which relate to stability and specific features but are not hierarchical or indicative of increasing injury severity. M1 corresponds to L5 transverse process avulsion fractures, signifying disruption of the iliolumbar ligaments. M2 pertains to displacement, and M3 identifies additional localised oedema visible on MRI, confirmed by CT. These modifiers can be applied individually or in combination.

## Figures and Tables

**Table 2:** List of outstanding questions on the management principles of the FFPs as a learning checklist for readers.

Theme	Open Questions	Suggestions and Existing Literature References
DIAGNOSTICS	What are the indications and optimal timing of advanced imaging, following the initial plain X-ray? Should each institution receiving patients with symptoms relevant to FFPs follow a specific diagnostic algorithm that will allow the prompt classification of these and formulation of an individualised care plan? Validation of the local diagnostic algorithm for the cost efficiency of different advanced imaging modalities (CT-scan, DECT, MRI scan). Do we need a diagnostic system for osteoporosis tailored specifically to obese patients?	Early employment of advanced imaging is highly recommended [34,35,37,38,39]. Development of specific diagnostic strategies should be relevant to the conditions of each institution/health system, aiming at a cost-efficient utilisation of resources [10,49]. Future studies should aim to compare the diagnostic accuracy of these modalities, considering their relevant cost and accessibility. The body weight and its effect on bone quality and fracture risk, as well as the challenge excessive body weight creates to the bone’s ability to withstand the force loads during a simple fall and those during mobilisation post FFPs [25,26].
ADMISSION MODEL	5. Should FFPs be admitted following the initial X-ray findings? 6. How do general hemostatic resuscitation protocols translate to the elderly population? 7. What should be the preferred model of care and admission speciality (geriatric or orthopaedic) for FFPs?	5. The need for monitoring of the hemodynamic status and safe post-injury mobilisation (under supervision) usually dictates a period of admission (observations for 24–48 h post-injury). 6. In older patients, monitoring the shock index (SI) is preferable to relying on traditional signs of hemodynamic instability. Balanced transfusion ratios and whole blood resuscitation show potential benefits. Tailored resuscitation protocols are preferable in this population [32]. 7. Development of local clinical pathways for FFPs can improve the timing and effectiveness of decision making, resulting in improvements in the provided care [10,48,49].
SURGICAL INDICATION	8. Validation of the different management strategies, as described by existing classification systems for FFPs. 9. Investigate potential differences between falls-related FFPs, insufficiency FFPs, and secondary fractures developing due to inadequate management of the FFPs.	8. Most researchers appear to have adopted the FFP classification and the AO Spine system for sacral fractures [46,76]. 9. Stratify surgical indications and management strategies according to these three different entities, often presented together as FFP.
MODE of FIXATION	10. Comparative analysis between different fixation strategies of the anterior and posterior ring, as well as of the effect of their complications. 11. Do we need patient-tailored fixation strategies and implants in specific categories (e.g., obesity, malignancy, presence of other implants, anatomic variations)?	10. Database analysis of large cohorts of patients may be more practical than conducting randomised prospective studies in this difficult-to-follow-up population [50,59,120,122]. 11. Database analysis of large cohorts, a result of multicenter collaboration, could potentially provide an answer to this question if we identify different outcomes in these different groups of FFPs.
AUGMENTATION STRATEGIES	12. What is the effect of the bone metabolic profile of the patients and/or of the antiosteoporotic treatment on the clinical outcome in different FFPs cohorts managed either nonoperatively or surgically? 13. Are augmentation methods practical, in which types of FFPs, at what anatomical regions, and with which injectable biomaterials?	12. Existing evidence supports the use of anabolic agents as well as supplementation therapies after testing bone turnover markers and serum minerals [10,109,110,111,112,113,114,115]. 13. Development of cost efficiency analysis on the use of augmentation methods to enhance fixation stability, promote unrestricted mobilisation, and accelerate FFP healing [100,101,104,105,106,107].
REHABILITATION TIMELINE & ENDPOINTS	14. What is a safe weight-bearing and mobilisation protocol for FFPs treated operatively or non-operatively? 15. Which are the most relevant patient outcome measures to the specific population of FFPs?	14. The aim should be to determine which of the different surgical methods facilitate unrestricted mobilisation as early as possible, and which of the FFPs can safely progress to that without surgical interventions [59,124,125]. 15. Develop patient-reported outcome and experience measures focusing on this particular population, and correlate these with standard quality of life, clinical, and radiological endpoints.

## Data Availability

Not applicable.
